# 

**Published:** 1907-09

**Authors:** 


### CONTENTS.

ORIGINAL COMMUNICATIONS—

The Venereal Peril. By H. McHatton, M. D.. Macon, Ga ---- 3[illegible]
The Treatment of Burns. By Frank K. Boland, M. D. Atlanta, Ga ---- 33[illegible]
A Visit to the Massachusetts State Sanatorium at Rutland. By Henry R. Slack, Ph. M., M. D., LaGrange, Ga 33[illegible]
A Plan for the Study of Man. By Arthur McDonald, Washington, D. C. ---- 336
Drainage of the Retention Catheter in Operations on the Genito-Urinary Tract. By L. Sage Hardin ---- 347
Particular Burns. By E. C. Clark. M. D.. Chapman, Ga ---- 849
Alcohol as a Cause of Disease. By N. P. Jelks ---- 352

CORRESPONDENCE ---- 355

EDITORIAL NOTES AND COMMENTS—

Trained Nurses ---- 358
Southern Medical Association ---- 359
Insurance Companies Return to the Five Dollar Fee ---- 360

NEWS AND NOTES ---- 361

BOOK REVIEWS ---- 364

SELECTIONS AND ABSTRACTS—

Pneumaturia ---- 366
Delayed Poisoning by Anesthetics ---- 366
Operations for Cleft Palate ---- 367
The Past, Present and Future of Tuberculosis ---- 368
A Method of Reducing Old Colles' Fractures ---- 368
Hemorrhoids and Their Treatment ---- 369
Transmissibility and Curability of Cancer ---- 371
Dr. McCormack to a Baltimore Audience ---- 371
A New Blood Test ---- 372
Tuberculin ---- 372
Penal Code State of New York ---- 873
The Therapeutics of Bright's Disease Based Upon Its Etiology ---- 375
The Reasons Why Physicians Succeed ---- 376
The Most Important Factor in Infant Mortality ---- 377
The Story of Rose and Edward ---- 379
Trautism as an Antiological Factor in Appendicitis ---- 382

